# Spermatogonial quantity in human prepubertal testicular tissue collected for fertility preservation prior to potentially sterilizing therapy

**DOI:** 10.1093/humrep/dey240

**Published:** 2018-07-25

**Authors:** J -B Stukenborg, J P Alves-Lopes, M Kurek, H Albalushi, A Reda, V Keros, V Töhönen, R Bjarnason, P Romerius, M Sundin, U Norén Nyström, C Langenskiöld, H Vogt, L Henningsohn, R T Mitchell, O Söder, C Petersen, K Jahnukainen

**Affiliations:** 1 NORDFERTIL Research Lab Stockholm, Department of Women's and Children's Health, Karolinska Institutet and University Hospital, Stockholm, Sweden; 2 Pediatric Endocrinology Unit, Department of Women’s and Children’s Health, Karolinska Institutet and University Hospital, Stockholm, Sweden; 3 Sultan Qaboos University, College of Medicine and Health Sciences, Muscat, Oman; 4 Reproductive Medicine, Department of Obstetrics and Gynaecology, Karolinska University Hospital, Stockholm, Sweden; 5 Department of Medicine, Karolinska Institutet, Stockholm, Sweden; 6 Clinic and University, Children's Medical Center, Landspítali University Hospital, Reykjavik, Iceland; 7 Faculty of Medicine, University of Iceland, Reykjavik, Iceland; 8 Department of Paediatric Oncology and Haematology, Clinical Sciences, Lund University, Lund, Sweden; 9 Division of Paediatrics, Department of Clinical Science, Intervention and Technology, Karolinska Institutet, Stockholm, Sweden; 10 Pediatric Blood Disorders, Immunodeficiency and Stem Cell Transplantation, Astrid Lindgren Children’s Hospital, Karolinska University Hospital, Stockholm, Sweden; 11 Clinical Sciences, Paediatrics, Umeå University, Umeå, Sweden; 12 Department of Paediatric Oncology, The Queen Silvia Children’s Hospital, Gothenburg, Sweden; 13 Department of Paediatrics, Faculty of Health Sciences, Linköping University, Linköping, Sweden; 14 Department of Clinical and Experimental Medicine, Faculty of Health Sciences, Linköping University, Linköping, Sweden; 15 Division of Urology, Institution for Clinical Science Intervention and Technology, Karolinska Institutet, Huddinge, Stockholm, Sweden; 16 MRC Centre for Reproductive Health, The Queen’s Medical Research Institute, The University of Edinburgh, Edinburgh, UK; 17 The Edinburgh Royal Hospital for Sick Children, Edinburgh, UK; 18 Department of Women’s and Children’s Health, Paediatric Oncology Unit, Karolinska Institutet, Stockholm, Sweden; 19 University Hospital, Stockholm, Sweden; 20 Division of Haematology-Oncology and Stem Cell Transplantation, Children´s Hospital, University of Helsinki, Helsinki University Central Hospital, Helsinki, Finland; 21 Department of Development and Regeneration, Organ System Cluster, Group of Biomedical Sciences, KU Leuven, Herestraat 49, Leuven, Belgium

**Keywords:** fertility preservation, childhood cancer, sickle cell disease, alkylating agents, spermatogonial quantity

## Abstract

**STUDY QUESTION:**

Does chemotherapy exposure (with or without alkylating agents) or primary diagnosis affect spermatogonial quantity in human prepubertal testicular tissue?

**SUMMARY ANSWER:**

Spermatogonial quantity is significantly reduced in testes of prepubertal boys treated with alkylating agent therapies or with hydroxyurea for sickle cell disease.

**WHAT IS KNOWN ALREADY:**

Cryopreservation of spermatogonial stem cells, followed by transplantation into the testis after treatment, is a proposed clinical option for fertility restoration in children. The key clinical consideration behind this approach is a sufficient quantity of healthy cryopreserved spermatogonia. However, since most boys with malignancies start therapy with agents that are not potentially sterilizing, they will have already received some chemotherapy before testicular tissue cryopreservation is considered.

**STUDY DESIGN, SIZE, DURATION:**

We examined histological sections of prepubertal testicular tissue to elucidate whether chemotherapy exposure or primary diagnosis affects spermatogonial quantity. Quantity of spermatogonia per transverse tubular cross-section (S/T) was assessed in relation to treatment characteristics and normative reference values in histological sections of paraffin embedded testicular tissue samples collected from 32 consecutive boy patients (aged 6.3 ± 3.8 [mean ± SD] years) between 2014 and 2017, as part of the NORDFERTIL study, and in 14 control samples (from boys aged 5.6 ± 5.0 [mean ± SD] years) from an internal biobank.

**PARTICIPANTS/MATERIALS, SETTING, METHODS:**

Prepubertal boys in Sweden, Finland and Iceland who were facing treatments associated with a very high risk of infertility, were offered the experimental procedure of testicular cryopreservation. Exclusion criteria were testicular volumes >10 ml and high bleeding or infection risk. There were 18 patients with a diagnosis of malignancy and 14 patients a non-malignant diagnosis. While 20 patients had the testicular biopsy performed 1–45 days after chemotherapy, 12 patients had not received any chemotherapy. In addition, 14 testicular tissue samples of patients with no reported testicular pathology, obtained from the internal biobank of the Department of Pathology at Karolinska University Hospital, were included as control samples in addition to reference values obtained from a recently published meta-analysis. The quantity of spermatogonia was assessed by both morphological and immunohistochemical analysis.

**MAIN RESULTS AND THE ROLE OF CHANCE:**

The main finding was a significant reduction in spermatogonial cell counts in boys treated with alkylating agents or with hydroxyurea for sickle cell disease. The mean S/T values in boys exposed to alkylating agents (0.2 ± 0.3, *n* = 6) or in boys with sickle cell disease and exposed to hydroxyurea (0.3 ± 0.6, *n* = 6) were significantly lower (*P* = 0.003 and *P* = 0.008, respectively) than in a group exposed to non-alkylating agents or in biobank control samples (1.7 ± 1.0, *n* = 8 and 4.1 ± 4.6, *n* = 14, respectively). The mean S/T values of the testicular tissue samples included in the biobank control group and the patient group exposed to non-alkylating agents were within recently published normative reference values.

**LIMITATIONS, REASONS FOR CAUTION:**

Normal testicular tissue samples included in this study were obtained from the internal biobank of Karolinska University Hospital. Samples were considered normal and included in the study if no testicular pathology was reported in the analysed samples. However, detailed information regarding previous medical treatments and testicular volumes of patients included in this biobank were not available.

**WIDER IMPLICATIONS OF THE FINDINGS:**

This study summarizes, for the first time, spermatogonial quantity in a prepubertal patient cohort just before and after potentially sterilizing treatments. Boys facing cancer and cytotoxic therapies are regarded as the major group who will benefit from novel fertility preservation techniques. There are no previous reports correlating spermatogonial quantity to cumulative exposure to alkylating agents and anthracyclines (non-alkylating agents) and no information about the timing of cytotoxic exposures among this particular patient cohort. For prepubertal boys in whom fertility preservation is indicated, testicular tissue should be obtained before initiation of chemotherapy with alkylating agents, whilst for those with sickle cell disease and treated with hydroxyurea, this approach to fertility preservation may not be feasible.

**STUDY FUNDING/COMPETING INTEREST(S):**

This study was supported by grants from The Swedish Childhood Cancer Foundation (PR2016-0124; TJ2016-0093; PR2015-0073, TJ2015-0046) (J.-B.S. and K.J.), the Jane and Dan Olssons Foundation (2016-33) (J.-B.S.), the Finnish Cancer Society (K.J.), the Foundation for Paediatric Research (J.-B.S.), Kronprinsessan Lovisas Förening För Barnasjukvård/ Stiftelsen Axel Tielmans Minnesfond, Samariten Foundation (J.-B.S.), the Väre Foundation for Paediatric Cancer Research (K.J.) and the Swedish Research Council (2012-6352) (O.S.). R.T.M. was supported by a Wellcome Trust Fellowship (09822). J.P.A.-L. and M.K. were supported by the ITN Marie Curie program ‘Growsperm’ (EU-FP7-PEOPLE-2013-ITN 603568). The authors declare no conflicts of interest.

**TRIAL REGISTRATION NUMBER:**

N/A.

## Introduction

Prepubertal boys treated with conditioning therapies for hematopoietic stem cell transplantation (HSCT) or testicular radiotherapy are known to be at very high risk of fertility impairment (Stukenborg *et al.*, 2018). Fertility preservation strategies for these boys primarily consist of cryopreservation of testicular tissue followed by auto-transplantation of spermatogonial stem cells (SSCs), tissue grafting or *in-vitro* maturation (Jahnukainen *et al.*, 2015; Picton *et al.*, 2015; Dalle *et al.*, 2017; McCracken and Nahata, 2017). However, no pregnancies have been so far reported with sperm obtained from these experimental strategies using immature testicular tissue (Picton *et al.*, 2015). The collection of testicular tissue is considered as an invasive procedure, and therefore the inclusion criteria for participating in testicular tissue cryopreservation programmes should be restricted to patients at the highest risk of infertility (i.e. those receiving total body irradiation or chemotherapy conditioning before HSCT, radiation applied to the testes or treatment with high dose alkylating agents) (Anderson *et al.*, 2015).

Only a small proportion of boys with malignancies will start therapy with agents that carry a high risk of infertility and therefore most boys are not offered cryopreservation initially. However, they may become eligible by fulfilling selection criteria, if further treatment is required due to poor response to therapy or relapse (Jahnukainen *et al.*, 2015). This means that the majority of patients eligible for fertility preservation have already received chemotherapy before testicular tissue cryopreservation is considered (Jahnukainen *et al.*, 2015; Balduzzi *et al.*, 2017). The previous studies reporting spermatogonial quantities in paediatric cohorts have mainly focused on archived historical material (Van Saen *et al.*, 2015; Poganitsch-Korhonen *et al.*, 2017) or report heterogonous material combining samples from boys with cancer and samples from boys with primary testicular pathology including Klinefelter syndrome and cryptorchidism (Heckmann *et al.*, 2018).

Today, various research teams have reported their experience of conducting fertility preservation programmes in prepubertal boys. This includes reports on sampling for cryobanking, patient and parent attitudes, and clinical decision factors for the parents and child (Anderson *et al.*, 2015; Picton *et al.*, 2015; Van Saen *et al.*, 2015; Heckmann *et al.*, 2018). However, to the best of our knowledge, there are no previous reports correlating spermatogonial quantity to cumulative exposure to cytotoxic drugs in a prepubertal patient cohort prior to potentially sterilizing oncological treatments. Therefore, information regarding the fertility status of testicular tissue obtained from prepubertal and pubertal boys subjected to gonadotoxic treatments before the time of biopsy is still very limited.

Regarding patients with non-malignant conditions who may be subjected to gonadotoxic treatments prior to biopsy, sickle cell disease (SCD) represents one of the largest groups worldwide in which allogeneic hematopoietic stem cell transplantation may be indicated. Patients with SCD suffer from vaso-occlusion that causes tissue hypoxia and organ damage. In order to reduce the incidence of vaso-occlusive crises, patients receive the antineoplastic agent hydroxyurea which prevents DNA synthesis through the inhibition of ribonucleoside diphosphate reductase. Therefore, these patients with SCD often receive treatment with hydroxyurea, and so a large proportion of boys with SCD facing HSCT and eligible for fertility preservation will have already received this chemotherapy before testicular tissue cryopreservation is offered (Jahnukainen *et al.*, 2015; Balduzzi *et al.*, 2017). Hydroxyurea is known to decrease sperm quality in adult males (Berthaut *et al.*, 2008); however, its effects on immature testes have not been described to date.

Fertility preservation in relapsed leukaemia and for patients with SCD demonstrates that, in clinical settings, it may be difficult to totally avoid exposure to cytotoxic therapies prior to testicular tissue cryopreservation. Therefore, information regarding the effect of previous exposure to potentially gonadotoxic treatment on spermatogonial quantity are required. Spermatogonial quantity per tubular cross section (S/T) has been shown to correspond to secretion of inhibin b, testosterone, luteinizing and follicle-stimulating hormone, and recently testicular reference values for S/T in human testes throughout healthy prepuberty have been established (Masliukaite *et al.*, 2016). In order to optimize patient selection and subsequent clinical cryobanking, these reference values were used to evaluate the effects of primary diagnosis and initial cancer treatment on spermatogonial quantity in the prepubertal testicular tissue collected for fertility preservation prior to potentially sterilizing therapy.

## Materials and methods

### Ethical approval

Ethical approval was obtained from the ethics Board of Karolinska Institutet and the Regional Ethics Board in Stockholm (Dnr 2013-2129-31-3, Dnr 2014/267-31/4 and Bio Bank approval BbK-01184), the National Ethics Board of Iceland, Reykjavik (VSN 15-002) and the Ethics Board of the University of Helsinki (426/13/03/03/2015).

### Patients and treatments

Prepubertal boys in Sweden, Finland and Iceland, who were facing treatments associated with a very high risk of infertility (allogeneic or autologous HSCT or testicular radiotherapy) (Skinner *et al.*, 2017), were offered the experimental procedure of testicular cryopreservation. Exclusion criteria were a high bleeding and/or infection risk. Inclusion criteria for the study were very high risk of therapy-related infertility and a testicular volume below 10 ml (measured by orchidometer). The parents and, where appropriate, the patient received verbal and written information about the research project and gave their written informed consent. Clinical treatment characteristics were recorded and pseudo-anonymised for entry into the research database. Patients underwent unilateral open testicular biopsy where less than 20% of the testicular volume of one testis was sampled. Two-thirds was cryopreserved according to a protocol published by Keros and colleagues for clinical fertility preservation (Keros *et al.*, 2007) and the remaining third was anonymized and transported to the NORDFERTIL research laboratory at Karolinska Institutet. Of 32 boys were enroled in the study from 2014 until 2017, 18 had a diagnosis of malignancy and 14 had a non-malignant diagnosis (Supplementary Table SI), while 20 had a testicular biopsy performed 1–45 days after a previous dose of chemotherapy. Alkylating agents were included in the therapeutic regimen for six patients while six SCD patients were treated with hydroxyurea, a chemotherapy agent. Alkylating agent exposures were calculated as mean cumulative cyclophosphamide equivalent doses (CEDs) (Green *et al.*, 2014) and cumulative doxorubicin were calculated as isotoxic dose equivalents (DIE) using conversion factor 1 for doxorubicin and 0.833 for daunorubicin. In addition, the daily dose of hydroxyurea (HU), the time since last exposure and as the time period between the start of chemotherapy and the biopsy are presented (Supplementary Table SI and Table I).

**Table I:** Descriptive statistics of testicular histology and treatment exposures for 32 boys who underwent testicular biopsy for fertility preservation and for 14 control samples from Karolinska Institutet’s internal biobank.

Parameter			Chemotherapy		
	Controls (biobank)	No chemotherapy	Hydroxyurea for SCD	Non-alkylating agents	Alkylating agents
	(*n* = 14)	(*n* = 12)	(*n* = 6)	(*n* = 8)	(*n* = 6)
Age (y)	5.6 ± 5.0	4.9 ± 3.1	7.9 ± 3.6	6.6 ± 4.8	7.3 ± 3.7
Spermatogonia/cross-section	4.1 ± 4.6	0.8 ± 0.9^*^	0.3 ± 0.6^*,##^	1.7 ± 1.0	0.2 ± 0.3^*,##^
Proportion of SCO tubules (%)	40 ± 30	69 ± 21^*,##^	90 ± 17^*,##^	46 ± 18	89 ± 14^*,##^
Exposure to cyclophosphamide (g/m^2^)	0	0	0	0	5.1 ± 3.2
CED (g/m^2^)	0	0	0	0	5.5 ± 3.0
Cumulative anthracycline dose (mg/m^2^)	0	0	0	161 ± 166	249 ± 118
Hydroxyurea (mg/kg)	0	0	24.5 ± 2.7	0	0

Abbreviations: CED, cumulative cyclophosphamide equivalent dose; SCO, Sertoli cell-only; SCD, sickle cell disease. ^*^P < 0.05 when compared to value in control group, ^##^*P* < 0.05 when compared to value in group exposed to non-alkylating chemotherapy.

In addition, 14 testicular samples (5.6 ± 5.0 [mean ± SD] years of age) without testicular pathology from the biobank of the Department of Pathology, Karolinska University Hospital served as controls.

### Tissue processing and histological analyses

Testicular biopsy samples from 46 boys (32 patients and 14 control testicular tissue samples) were fixed in formalin, 4% in phosphate buffered saline and afterwards embedded in paraffin. Section (5 μm thick) were obtained and non-consecutive sections, randomly selected, were stained with periodic acid-Schiff (PAS) or immunostained with an antibody against a germ cell marker (DDX4) as previously described (Alves-Lopes *et al.*, 2017). In brief, following antigen retrieval, sections were blocked with a blocking buffer containing normal donkey serum 10% (NDS; 017-00-121, Jackson Immuno Research, PA, USA) and 1.5% bovine serum albumin (BSA) in Tris-buffered saline (TBS) at room temperature for one hour. Sections were incubated at 4°C overnight with mouse monoclonal anti-DDX4 primary antibody (ab27591, Abcam, Cambridge, UK; final concentration 1.7 μg/ml) and rabbit polyclonal anti-SOX9 (AB5535, Merck Millipore, Frankfurt, Germany, final concentration 4.3 μg/ml) diluted in blocking buffer. For negative controls, primary antibodies were replaced by mouse IgGs (sc-2025, Santa Cruz Biotechnology, Dallas, TX, USA; final concentration 4 μg/ml) and rabbit IgGs (ab 27478, Abcam, Cambridge, UK; final concentration 2 μg/ml) diluted in blocking buffer (TBS/NDS/BSA). Sections were washed in TBS three times for 5 min each, followed by incubation for 1 h at room temperature with Alexa 488 donkey anti-mouse (715546150, Thermo Fisher, Waltham, USA; final concentration 3 μg/ml) and Cy3-conjugated donkey anti-rabbit (11483299, Thermo Fisher; final concentration 3 μg/ml) secondary antibodies diluted in blocking buffer. Sections were washed in TBS three times for 5 min each and mounted in VECTASHIELD mounting medium with DAPI (H-1500, Vector, CA, USA),

PAS kit (101646, Merck, Darmstadt, Germany) was used to stain the sections according to the manufacturer’s protocol. Briefly, after re-hydration, sections were washed in distilled water twice for 5 min each and incubated in periodic acid for 5 min. This was followed by thorough washing under running tap water and then twice in distilled water for 5 min each. Afterwards, sections were incubated in Schiff’s reagent for 15 min and washed thoroughly again under running tap water and twice in distilled water for 5 min each. Then, samples were counter-stained with hematoxylin (Mayer’s Hemalaun solution, 1092491000, Merck) for 2 min and washed in running tap water for 2 min, dehydrated and finally mounted in Entellan® new (1079610100, Merck).

Blind analysis of spermatogonial numbers per round tubular cross-section (S/T) was performed by an experienced examiner using a bright-field microscope (Eclipse E800, Nikon; Japan). All or, at least 25, round tubular cross-sections per sample were evaluated to achieve result validity (Stadtler and Mausle, 1971). The spermatogonia were identified in non-consecutive tissue sections from each patient on the basis of their morphology (size, shape and location) (Paniagua and Nistal, 1984) in PAS stained sections as well as on the basis of their DDX4 expression (Supplementary Fig. S1). There was a minimum distance of at least 15 μm between sections used for each staining method from the same patient sample to avoid the evaluation of identical cells by the two staining methods. For spermatogonial quantification, a significant correlation (*r* = 0.828, *P* < 0.0001; Supplementary Fig. S2) existed between morphology and immunostaining, and therefore only results from DDX4-staining are presented.

### Statistical analysis

Results are reported as mean ± standard deviation (SD) and range. Student’s unpaired *t* test (normally distributed variables), the Mann–Whitney *U* test (non-normally distributed variables) and Pearson correlation coefficient (*r*) were used as appropriate to determine the relationships between spermatogonial quantity, age and treatment characteristics. Analyses were performed using IBM SPSS Statistics V23.0 software (IBM Corporation, Armonk, NY, USA).

## Results

### Quantity of spermatogonia per seminiferous tubule in immature testes of boys

**Figure 1:**
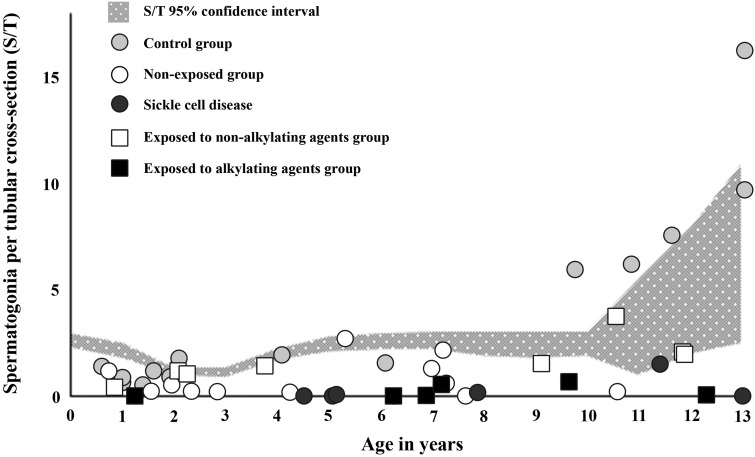
Spermatogonial numbers per tubular cross-section (S/T) of testicular tissues obtained for fertility preservation from boys with sickle cell disease receiving hydroxyurea (black circles), those exposed to alkylating (black squares) or non-alkylating chemotherapy (white squares), and those not exposed to previous treatment (white dots) as well as biobank controls (light grey dots). Each dot or square represents the mean spermatogonial number for an individual patient. The *Y*-axis represents patient age in years. Data are plotted on a meta-regression fit line of reference values for spermatogonia per tubular cross section (S/T) throughout healthy prepuberty, as previously reported (Masliukaite *et al.*, 2016).

Mean S/T in samples from cancer patients exposed to non-alkylating agents (1.7 ± 1.0, *n* = 8) and biobank controls (4.1 ± 4.6, *n* = 14) did not exhibit significant differences in S/T numbers. Both groups were within the 95% confidence intervals of normative reference values (Masliukaite *et al.*, 2016) (Table I, Fig. 1). In contrast, samples from cancer patients exposed to alkylating agents, exhibited a lower mean S/T value (0.2 ± 0.3, *n* = 6) compared with samples from patients treated with non-alkylating agents (*P* = 0.003) or biobank controls (*P* < 0.001) (Table I, Fig. 1). Spermatogonial quantity correlated with cumulative exposure to alkylating agents (*r* = −0.579, *P* = 0.024), but not to anthracyclines (*r* = −0.133, *P* = 0.637) or patient age (*r* = 0.398, *P* = 0.142). Among five boys exposed to CED close to or over 4000 mg/m^2^, S/T values were close to zero (Supplementary Table SI). In samples from six patients with SCD, a lower mean S/T value (0.3 ± 0.6, *n* = 6) was found compared with samples from biobank controls (*P* = 0.003) (Table I, Fig. 1). Five boys with SCD aged 4–7 and 13 years had a totally depleted spermatogonial pool, while the remaining 11.5 year old boy had low spermatogonial quantity compared to normal values present in the control material (Fig. 1, Supplementary Table SI). All boys with SCD had received hydroxyurea.

The patients with malignant or non-malignant diagnoses and who were not previously exposed to chemotherapy showed marked variation in their spermatogonial quantity (Fig. 1, Supplementary Table SI). Five patients with known single gene mutations (three thalassaemia majors, one Fanconi anaemia and one immunodeficiency caused by variant of FOXP3-gene) showed reduced spermatogonial quantity (0.4 ± 0.5) compared to controls (*P* = 0.006) (Supplementary Table SI).

## Discussion

Although, fertility preservation in boys before or after gonadotoxic medical treatments is currently a hot topic in the field of reproductive medicine/biology and paediatric oncology/haematology, there are no previous reports that correlate spermatogonial quantity with cumulative exposure to alkylating agents and anthracyclines or to information about timing of cytotoxic exposures among this particular patient cohort. This study describes, for the first time, effects of previous cytotoxic therapy on spermatogonial quantity in a prepubertal patient cohort just before potentially sterilizing oncological treatments and fertility preservation. The main finding was that boys receiving alkylating agents and SCD patients receiving hydroxyurea had significantly depleted numbers of spermatogonia in their testicular tissue samples compared to other patients exposed to non-alkylating agents or to biobank controls.

In a recent retrospective study evaluating testicular tissue samples obtained between 1979 and 1995 (Poganitsch-Korhonen *et al.*, 2017), a depletion of the spermatogonial pool was demonstrated after a CED above 4000 mg/m^2^. Despite the limited number of samples, similarities in quantity of spermatogonia per transverse tubular cross-section (S/T) were observed in comparable patient groups between the two studies. For non-alkylating exposed boys, mean SD values for S/T were 1.7 ± 1.0 in this study, compared with 1.6 ± 0.8 in the previous study (Poganitsch-Korhonen *et al.*, 2017), whilst for boys exposed to alkylating agents mean SD values for S/T were 0.2 ± 0.3 and 0.4 ± 0.5, respectively. Mean age across all groups in the two studies ranged from 5.3 to 7.3 years. In the study of Poganitsch-Korhonen and colleagues, the number of spermatogonia was compared to reference values obtained from a meta-analysis published in 2016 (Masliukaite *et al.*, 2016). The reference values summarized the quantity of spermatogonia in boys up to the age of 14 years. A decreasing number of spermatogonia per seminiferous tubular cross section from 2.5 to 1.2 was described from birth to three years of age, followed by an increase up to 2.6 spermatogonia per tubular cross section at the age of six-to-seven and a plateau until the age of eleven. At the onset of puberty an incline up to seven spermatogonia per tubular cross section could be observed (Masliukaite *et al.*, 2016).

Our observations on spermatogonia quantity in boys exposed to treatment protocols used between 2014 and 2017 are in concordance with our previous study on tissue samples of boys treated between 1978 and 1995 (Poganitsch-Korhonen *et al.*, 2017) as well as previous reports showing that leukaemia therapy in childhood involving alkylating agents results in depletion of the spermatogonial pool and decreased fertility after cumulative CEDs greater than 4000 mg/m^2^ (Nurmio *et al.*, 2009; Green *et al.*, 2014). Therefore, these results strongly suggest that tissue samples containing SSCs for fertility preservation should be collected before initiating alkylating agent therapies.

The second main outcome of this study is that spermatogonial quantity is significantly reduced in testes of prepubertal boys treated with hydroxyurea for SCD. Our findings demonstrated a highly significant reduction, or in some cases total depletion, of spermatogonia in SCD patients treated with hydroxyurea. A previous case study reported a 29 year old SCD patient with a normal sperm count (88 × 10^6^ sperm/ml) prior to treatment, who became azoospermic one month after commencing hydroxyurea (Garozzo *et al.*, 2000). Ten months after his hydroxyurea treatment was discontinued, his sperm count had partially recovered (35 × 10^6^ sperm/ml), suggesting a reversible effect on sperm counts in adult men receiving hydroxyurea (Garozzo *et al.*, 2000). This study, was followed by a retrospective study investigating the influence of clinically relevant hydroxyurea (20–30 mg/kg body weight/day) on fertility in adult male SCD patients (Berthaut *et al.*, 2008). This study by Berthaut and colleagues demonstrated a negative effect of hydroxyurea on semen parameters during treatment, which persisted in some of the patients after cessation of treatment (Berthaut, *et al.* 2008). Another study, addressing gonadal function in children with SCD (2.25–14.16 years of age) and not exposed to hydroxyurea, revealed spontaneous puberty in boys after HSCT (Brachet *et al.*, 2004). However, boys included in this study had smaller testes and increased serum levels of follicle-stimulating hormone, suggesting an impaired germinal epithelium, which may have resulted from HSCT (Brachet *et al.*, 2004). One previous study has evaluated the quality of testicular tissue from SCD boys and observed depletion of spermatogonia in two out of five boys aged between 5 and 15 years (Van Saen *et al.*, 2015). SCD itself can decrease spermatogonial quantity, since vaso-occlusions and chronic anaemia are associated with decreased pubertal testicular growth (Martins *et al.*, 2015). The SCD boys in the present study were all exposed to hydroxyurea (20–25 mg/kg) which is an S-phase-specific cytotoxic and antineoplastic agent (Wang *et al.*, 2011). Hydroxyurea is reported to suppress spermatogenesis in adult men with SCD (DeBaun, 2014). The present findings suggest that it may also cause spermatogonial loss before puberty, although the relative contribution of the disease or the treatment remains to be established. In the present study detailed identification of exact treatment related effects could not be separated from the direct effects of the SCD itself. Therefore, further studies in boys with SCD comparing those exposed to hydroxyurea with non-exposed SCD patients are needed before final recommendations regarding fertility preservation in this patient population can be given.

In addition to reduced numbers of spermatogonia in SCD patients treated with hydroxyurea, testicular tissue samples obtained from boys diagnosed with other single gene mutations (thalassaemia majors, Fanconi anaemia and immunodeficiency caused by variant of FOXP3-gene) showed a reduced quantity of spermatogonia. These observations suggest that genetic abnormalities in haematological diseases may also be associated with reduced numbers of spermatogonia in prepubertal testes. The spermatogonial quantity in these patients prior to the initiation of any chemotherapy may reflect the effects of genetic diagnosis and constitutional disease on testicular development and fertility potential.

However, a limitation of our study was that the internal biobank did not provide information about the medical treatment of subjects prior to tissue sampling. This could affect the validity of this population as a control group. Therefore, in order to validate the biobank tissues as appropriate control material, we compared the samples to the normative reference values obtained from a meta-analysis of six independent studies (Masliukaite *et al.*, 2016). This demonstrated that the biobank control material was within the previously published normative reference values.

In conclusion, we have demonstrated that spermatogonial quantity in clinical fertility preservation material collected after cancer therapy does not differ from biobank control samples and remains within normative reference values for prepubertal boys who did not receive alkylating agents. However, for those boys receiving alkylating agents and SCD patients receiving hydroxyurea there is a significant depletion of the spermatogonial pool when compared to the biobank control samples. Therefore, in order to collect sufficient amounts of spermatogonial stem cells for intended fertility preservation, a testicular biopsy sample should be acquired prior to exposure to alkylating agents. Boys with SCD and their parents should be aware of the predisposition for reduced spermatogonial quantity and decreased potential for successful fertility preservation as a result of the disease and/or the exposure to hydroxyurea therapy.

## Supplementary Material

Supplementary Figure 1

Supplementary Figure 2

Supplementary Table 1
