# Supplementary material for: Spermatogonial quantity in human prepubertal testicular tissue collected for fertility preservation prior to potentially sterilizing therapy
Source: Hum Reprod. 2018 Jul 25;33(9):1677–83. doi: 10.1093/humrep/dey240 (PMC6112575; doi:10.1093/humrep/dey240)
Supplement: Supplementary Figure 1 [file dey240suppl_figure1.pdf]

Human Reproduction, 2018
doi:10.1093/humrep/dey240

human reproduction

SUPPLEMENTARY DATA

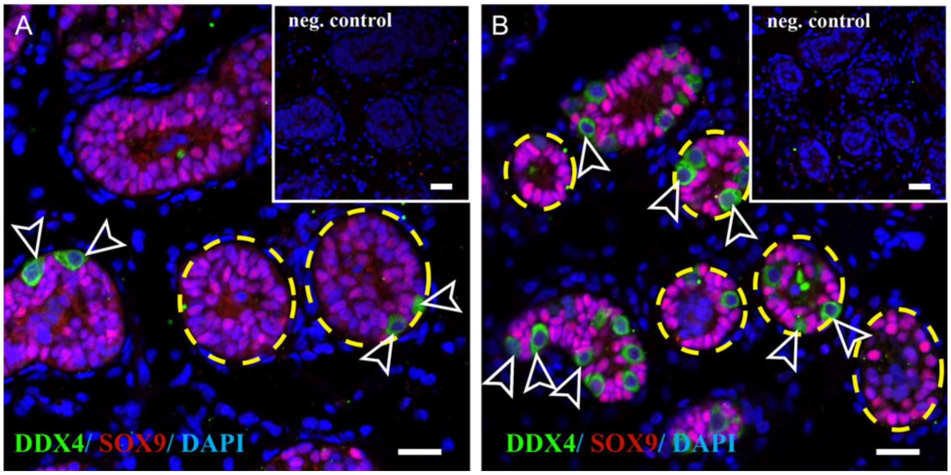

Supplementary Figure S1 Expression of DDX4 (arrowheads: germ cell marker; green staining) and SOX9 (Sertoli cell marker; red staining) shown in testicular tissue of boys aged 0.7 and 9.2 years and diagnosed with JMML (no chemotherapy treatment) and MDS (exposed to non-alkylating agents), respectively. Counterstain with DAPI (blue staining); negative controls with rabbit IgGs instead of the primary antibodies are shown as insets for each staining. Dashed yellow lines delineate tubules included in the evaluation of germ cells per tubular cross-section. Scale bars: 50 µm.
