# Supplementary material for: Spermatogonial quantity in human prepubertal testicular tissue collected for fertility preservation prior to potentially sterilizing therapy
Source: Hum Reprod. 2018 Jul 25;33(9):1677–83. doi: 10.1093/humrep/dey240 (PMC6112575; doi:10.1093/humrep/dey240)
Supplement: Supplementary Figure 2 [file dey240suppl_figure2.pdf]

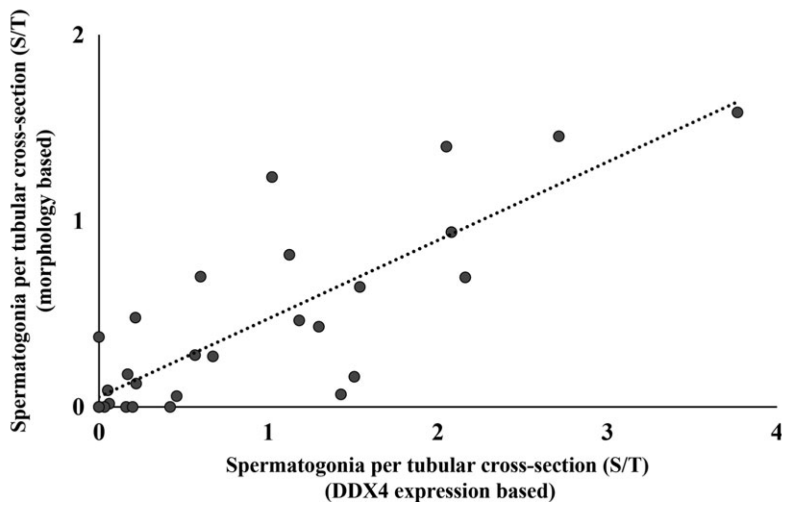

Supplementary Figure S2 Correlation between spermatogonial numbers, based on morphology or DDX4 expression, per tubular cross-section (S/T) in testicular tissues obtained for fertility preservation. Data from 32 boys, including those with sickle cell disease receiving hydroxyurea, patients receiving alkylating or non-alkylating chemotherapy and those not exposed to previous treatment, were evaluated.
