# Supplementary material for: Spermatogonial quantity in human prepubertal testicular tissue collected for fertility preservation prior to potentially sterilizing therapy
Source: Hum Reprod. 2018 Jul 25;33(9):1677–83. doi: 10.1093/humrep/dey240 (PMC6112575; doi:10.1093/humrep/dey240)
Supplement: Supplementary Table 1 [file dey240suppl_table1.pdf]

Supplementary Table SI **Patient and treatment characteristics for 32 boys who underwent testicular biopsy for fertility preservation.**

Group	No	Diagnosis	Age (y)	CED (mg/m^2)	DIE (mg/m^2)	HU (mg/kg)	Initiation of first treatment before biopsy (days)	Last treatment before biopsy (days)	Testis volume (ml)	S/T
Control (biobank samples)										
	1	N/A	0.6	N/A	N/A	N/A	N/A	N/A	N/A	1.40
	2	N/A	1.0	N/A	N/A	N/A	N/A	N/A	N/A	0.65
	3	N/A	1.0	N/A	N/A	N/A	N/A	N/A	N/A	0.88
	4	N/A	1.4	N/A	N/A	N/A	N/A	N/A	N/A	0.56
	5	N/A	1.6	N/A	N/A	N/A	N/A	N/A	N/A	1.15
	6	N/A	1.9	N/A	N/A	N/A	N/A	N/A	N/A	0.90
	7	N/A	2.1	N/A	N/A	N/A	N/A	N/A	N/A	1.75
	8	N/A	4.1	N/A	N/A	N/A	N/A	N/A	N/A	1.95
	9	N/A	6.1	N/A	N/A	N/A	N/A	N/A	N/A	1.56
	10	N/A	9.8	N/A	N/A	N/A	N/A	N/A	N/A	5.99
	11	N/A	10.9	N/A	N/A	N/A	N/A	N/A	N/A	6.24
	12	N/A	11.7	N/A	N/A	N/A	N/A	N/A	N/A	7.61
	13	N/A	13.1	N/A	N/A	N/A	N/A	N/A	N/A	9.76
	14	N/A	13.1	N/A	N/A	N/A	N/A	N/A	N/A	16.37
No chemotherapy										
	15	JMML	0.7	0	0	0	NT	NT	1	1.18
	16	PID	1.6	0	0	0	NT	NT	2	0.22
	17	PID	2.0	0	0	0	NT	NT	2	0.46
	18	Thalassaemia major	2.4	0	0	0	NT	NT	2	0.22
	19	Neuroblastoma	2.9	0	0	0	NT	NT	<10	0.23
	20	Thalassaemia major	4.3	0	0	0	NT	NT	1–2	0.17
	21	Adrenoleukodystrophy	5.3	0	0	0	NT	NT	<10	2.71
	22	Thalassaemia major	7.0	0	0	0	NT	NT	2	1.30
	23	MDS	7.2	0	0	0	NT	NT	<10	2.16
	24	AML	7.3	0	0	0	NT	NT	<10	0.60
	25	Fanconi anaemia	7.7	0	0	0	NT	NT	1	0.00
	26	SAA	10.6	0	0	0	NT*	NT*	<10	0.20
Sickle Cell Disease										
	27	SCD	4.5	0	0	26	N/A	1	<10	0.00
	28	SCD	5.1	0	0	24.4	N/A	1	2	0.00
	29	SCD	5.2	0	0	20	N/A	1	2	0.06
	30	SCD	7.9	0	0	27.4	N/A	1	<10	0.16
	31	SCD	11.5	0	0	23	N/A	18	2	1.51
	32	SCD	13.1	0	0	26.4	N/A	1	<10	0.00
Chemotherapy with non-alkylating agents										
	33	JMML	0.8	0	0	0	63	>45	<10	0.42
	34	Hepatoblastoma	2.1	0	450	0	17	3	<10	1.13
	35	PNET	2.3	0	0	0	26	24	<10	1.02
	36	AML	3.8	0	300	0	91	28	2	1.43
	37	MDS	9.2	0	80	0	>1622	>3	<10	1.54
	38	ALL	10.6	0	80	0	54	18	<10	3.77

Continued

Supplementary Table SI *Continued*

Group	No	Diagnosis	Age (y)	CED (mg/m²)	DIE (mg/m²)	HU (mg/kg)	Initiation of first treatment before biopsy (days)	Last treatment before biopsy (days)	Testis volume (ml)	S/T
	39	AML	11.9	0	300	0	102	23	<10	2.08
	40	ALL	11.9	0	80	0	32	2	<10	2.05
Chemotherapy with alkylating agents										
	41	Neuroblastoma	1.3	10 500	250	0	262	12	<10	0.00
	42	Wilms tumour	6.3	4814	200	0	338	6	<10	0.00
	43	ALL	6.9	4400	160	0	166	22	<10	0.03
	44	ALL	7.2	7600	480	0	956	14	2	0.57
	45	ALL	9.7	2000	180	0	1986	24	2–3	0.67
	46	ALL	12.4	3976	225	0	865	14	1	0.05

Abbreviations: CED, cumulative cyclophosphamide equivalent dose; DIE, doxorubicin isotoxic dose equivalent; HU, hydroxyurea; S/T, spermatogonia per seminiferous tubular cross section; NT, no treatment; PID, primary immunodeficiency; JMML, juvenile myelomonocytic leukaemia; MDS, myelodysplastic syndrome; SCD, Sickle cell disease; ALL, acute lymphoblastic leukaemia; AML, acute myeloid leukaemia; PNET Primitive neuroectodermal tumour; SAA, severe aplastic anaemia; N/A, no information available. *Long-term treatment with cyclosporin 1.9 mg/kg until one day before biopsy.
